# Supplementary material for: A convergent malignant phenotype in B-cell acute lymphoblastic leukemia involving the splicing factor SRRM1
Source: NAR Cancer. 2022 Dec 9;4(4):zcac041. doi: 10.1093/narcan/zcac041 (PMC9732526; doi:10.1093/narcan/zcac041)
Supplement: zcac041_Supplemental_Files [file zcac041_supplemental_files.zip › supp file description of data files v23.1.docx]

**Description of data files**

**Data file 1. Table with detailed clinical information for all patients obtained from the original sources**. *Sample_ID* is the code used to identify the sample. *Patient_ID* is the code used to identify the patient. Project indicates the sample cohort of origin. *Type* indicates the type of leukemia. *Tissue* indicates the origin of the sample. *Blasts* indicates the proportion of blasts in the sample. DX_RL indicates the time when the sample was obtained at diagnosis (DX) or after relapse (RL). *Gender* indicates whether the patient is female (F) or male (M). *Age* indicates the age of the patient in months. *Fusions* indicates the fusion recorded in the clinical information. *Cell.of.Origin* indicates if the samples were obtained from B-cells or B-cells precursors. *First.event* indicates if the patient had a relapse or not. *Event.Free.survival.time.in.days* is the time in days when the first event appeared. *Vital.Status* indicates if the patient is dead or not. *Overall.Survival.Time.in.Days* is the time until the patient's death or the last time of follow-up.

**Data file 2. A manually curated list of RBPs and splicing factors used in the manuscript.**

**Data file 3. Table with all fusions detected.** Columns indicate sample code (*Sample*), patient ID (*ID*), cohort, source of the fusion calling (Star-fusion, Clinical information, or both), cancer type in which the fusion has been observed before (*Cancer_category*), fusion group in which the fusion has been assigned (Group), number of reads supporting the fusion junction (*JunctRC*), number of spanning reads supporting the fusion (*SpanRC*), expression of the fusion in Fusion Fragments per Million (*FFPM*), Ensembl gene identifier of the first fusion gene (*ENG1*), breakpoint coordinates of the first fusion gene (*BP1*), Ensembl gene identifier of the second fusion gene (*ENSG2*), breakpoint of the second fusion gene (*BP2*), type of chromosomal rearrangement (annotation), FAF of the first fusion gene (*FAF_gene1*), FAF of the second fusion gene (*FAF_gene2*), and combined FAF (*FAF*).

**Data file 4. Tables with all differential expression analyses between fusion groups.** The comparisons and labels correspond to those shown in supplementary figure 9. The GeneID column indicates the gene name. LogFC is the log2-fold-change between the two conditions. AveExpr is the average log2-expression for the gene over all samples. T is the moderated t-statistic. P.Value is the raw p-value. Adj.P.Val is the adjusted p-value using the BH method. B is the log-odds that the gene is differentially expressed.

**Data file 5. Differential expression analysis between *KMT2A*-r and *ETV6*-r patients.** The GeneID column indicates the gene name. LogFC is the log2-fold-change between the two conditions. AveExpr is the average log2-expression for the gene over all samples. T is the moderated t-statistic. P.Value is the raw p-value. Adj.P.Val is the adjusted p-value using the BH method. B is the log-odds that the gene is differentially expressed.

**Data file 6. Pearson correlation for the signature genes with the risk score.** Column *statistic* is the value of the test statistic, *p.value* is the p-value of the test and *estimate* is the R of the correlation test.

**Data file 7. Signature scores for all the patients and cell lines studied. Column *Sample* indicates the sample code.** Column *None* indicates the score associated with not having a relapse. Column *Relapse* indicates the score related to not having a relapse. Column *Sample_type* indicates if the origin of the sample is a patient at the diagnostic stage (Diagnostic), a patient at relapse stage (Relapse), a cancer cell line (CCLE), or a normal sample (GM12878 cell line, B-cell progenitors).

**Data file 8. Differential splicing events between high and low-risk patients.** Columns indicate the event ID, dPSI, p-value, and FDR-adjusted p-value as calculated with the linear model (see Methods), HGNC gene symbol, transcripts supporting the inclusion of the event (Alt 1), transcripts supporting the exclusion of the event (Alt 2), column indication whether any of Alt 1 or Alt 2 transcripts are annotated as coding (transcript_type), cancer hallmarks associated to the differentially spliced genes (hallmarks), fusions in which the event shows co-occurrence with high risk (high_risk_cooccur) and presence of motifs for SRRM1 interacting RBPs(SRRM1_RBPs_motifs).

**Data file 9. Differential splicing events between *KMT2A*-r and *ETV6*-r patients.** Columns indicate the event IDs, dPSI, p-value, and FDR-adjusted p-value as calculated with the linear model (see Methods).

**Data table 10. Differential splicing events between B-cell precursors and GM12878 cells.** Columns indicate the event ID, dPSI, and p-value provided by SUPPA. Events for which the method could not retrieve a dPSI value were omitted (see Methods).
